# Antitumor efficacy of cytosine deaminase-armed vaccinia virus plus 5-fluorocytosine in colorectal cancers

**DOI:** 10.1186/s12935-020-01340-6

**Published:** 2020-06-15

**Authors:** Yuedi Ding, Jun Fan, Lili Deng, Biao Huang, Bin Zhou

**Affiliations:** 1 NHC Key Laboratory of Nuclear Medicine, Jiangsu Key Laboratory of Molecular Nuclear Medicine, Jiangsu Institute of Nuclear Medicine, Wuxi, 214063 Jiangsu China; 2 Department of Radiopharmaceuticals, School of Pharmacy, Nanjing Medical University, Nanjing, 211166 China

**Keywords:** Vaccinia virus, Cytosine deaminase, Antitumor efficacy, Colorectal cancer

## Abstract

**Background:**

Vaccinia viruses have emerged as attractive therapeutic candidates for cancer treatment due to their inherent ability of tumor tropism and oncolytic property. Cytosine deaminase (CD), which is derived from bacteria or yeast, can convert a relatively nontoxic prodrug 5-fluorocytosine (5-FC) into the active anticancer drug 5-Fluorouracil (5-FU). Vaccinia virus armed with the prodrug-activator CD gene would result in augmented antitumor effects that combined the effect of vaccinia virus and 5-FU together, and particularly limited the anticancer drug to tumor regions.

**Methods:**

The attenuated vaccinia Tian Tan strain Guang 9 (VG9), with active yeast CD expression and thymidine kinase (TK) deficiency, was successfully constructed. Then, in vitro and in vivo antitumor efficacy of vaccinia VG9-CD plus 5-FC administration was evaluated in colorectal cancer cells.

**Results:**

Vaccinia viruses displayed different oncolytic potency in vitro cells, no relationship with whether they were cancer cells or normal cells. In colorectal tumor models, mice treated with vaccinia VG9-TK^−^ showed better tumor remission ability and prolonged survival. Moreover, vaccinia VG9-CD in combination with gavage administration of 5-FC displayed the best antitumor efficacy, especially for the prolongation of survival.

**Conclusions:**

Vaccinia VG9-CD in combination with 5-FC plays combined effect of vaccinia virus and chemotherapy, and becomes a promising virotherapy for cancer.

## Background

As the third most commonly diagnosed cancer in males and the second in females, colorectal cancer has an estimated 1.4 million cases and 693,900 deaths occurring in 2012 [1–4]. A number of therapeutic methods have been applied to colorectal cancer, surgical excision is an effective method to remove cancer lesions, cytotoxic drug are used as a first line of defense [5–7]. However, tumor metastases and drug resistance are the principal obstacles in the treatment of colorectal cancer. Therefore, novel approaches are required to improve the effectiveness of chemotherapy and prevent tumor recurrence after surgery [8–11]. Oncolytic virotherapy is an attractive therapeutic option as it has great promise to eradicate tumors with minimal toxicity to healthy tissues [12]. Furthermore, oncolytic viruses prevent tumor recurrence via the induction of long-term immunological memory [13].

Vaccinia virus Tian Tan strain, which historically used for the vaccination of smallpox prevention, led to the eradication of Variola in China prior to 1980 [14–16]. Now, vaccinia viruses have emerged as attractive therapeutic candidates for cancer treatment due to their inherent ability of tumor tropism and oncolytic property [17, 18]. Thymidine kinase (TK) in vaccinia virus, is an enzyme needed for nucleic acid metabolism. Deletion of the TK gene inhibits viral replication in normal non-dividing cells, however, tumor cells have an increased pool of functional nucleotides allowing vaccinia virus replication in the absence of viral TK [19]. Therefore, genetic insertion of therapeutic transgenes into the TK region, could express exogenous therapeutic proteins within the confines of the tumor, which lead to enhanced antitumor efficacy [20].

5-Fluorouracil (5-FU), one of the most commonly used chemotherapeutic drugs in cancer therapy, has the limitations of short half-life, lack of selectivity for tumor cells and the appearance of drug resistance [21, 22]. Cytosine deaminase (CD), derived from bacteria or yeast, can convert a relatively nontoxic prodrug 5-fluorocytosine (5-FC) into the active drug 5-FU [23, 24]. Arming vaccinia virus with a prodrug-activator gene might result in augmented antitumor effects that combined the effect of vaccinia virus and chemotherapy together, but with less systemic toxicity [25]. 5-FC is an orally bioavailable FDA approved antifungal drug that efficiently crosses the blood–brain barrier [22]. CD-armed vaccinia virus, in conjunction with subsequent 5-FC, provides direct killing of tumor cells by local production of 5-FU. The vaccinia virus also induced a local and systemic immunotherapeutic response resulting in long-term survival after cessation of 5-FC treatment [26, 27].

Vaccinia virus Guang9 (VG9), which was derived from Vaccinia virus Tian Tan strain by using a traditional single plaque purification method, is an attenuated strain that mediates antitumor effects as both an oncolytic agent and a viral vector for therapeutic gene delivery [28, 29]. Tumor selectivity and immunity of TK-deleted VG9 have been validated in previously study [17, 18]. Now, an optimized yeast CD gene inserted into the TK region of VG9 was constructed to express active yeast CD protein and effectively converted the prodrug 5-FC into the anticancer drug 5-FU. Then, the in vitro and in vivo antitumor efficacies of CD-armed VG9 combined with 5-FC administration were assessed in colorectal cancer models.

## Materials and methods

### Drug and reagents

5-FC, 5-FU, xanthine and hypoxanthine were purchased from Sigma-Aldrich. Mycophenolic acid was purchased from Sangon Biotech. Fluorocytosine tablets produced by China Pharmaceutical University (CPU)-Pharma were ordered for in vivo assays.

### Cell lines

The colon adenocarcinoma cell line HCT116 (human) and CT26.WT (murine) were originally obtained from Cell Library of Biochemistry and Cell Biology, CAS. The MC38 murine colon adenocarcinoma cancer cells were originally obtained from Cell Resource Center, Peking Union Medical College, NSTI. HCT116 cells were cultured in Dulbecco’s modified eagle medium (Life Technologies, catalog #12,100) containing 10% fetal bovine serum (FBS). CT26 and MC38 cell lines were maintained in RPMI 1640 medium (Life Technologies, catalog #23,400) supplemented with 10% FBS. Cells were maintained in a humidified atmosphere with 5% CO_2_ at 37 °C.

### Mice

BALB/c nu, C57BL/6 and BALB/c mice were provided by Cavens Laboratory Animal (Changzhou, China). All animals were maintained under specific pathogen-free conditions; all animal procedures were conducted in accordance with the Laboratory Animal-Guideline of welfare ethical review of Chinese Institutional Animal Care and Use Committee (IACUC).

### Construction of recombinant vaccinia VG9-CD

**Fig. 1:**
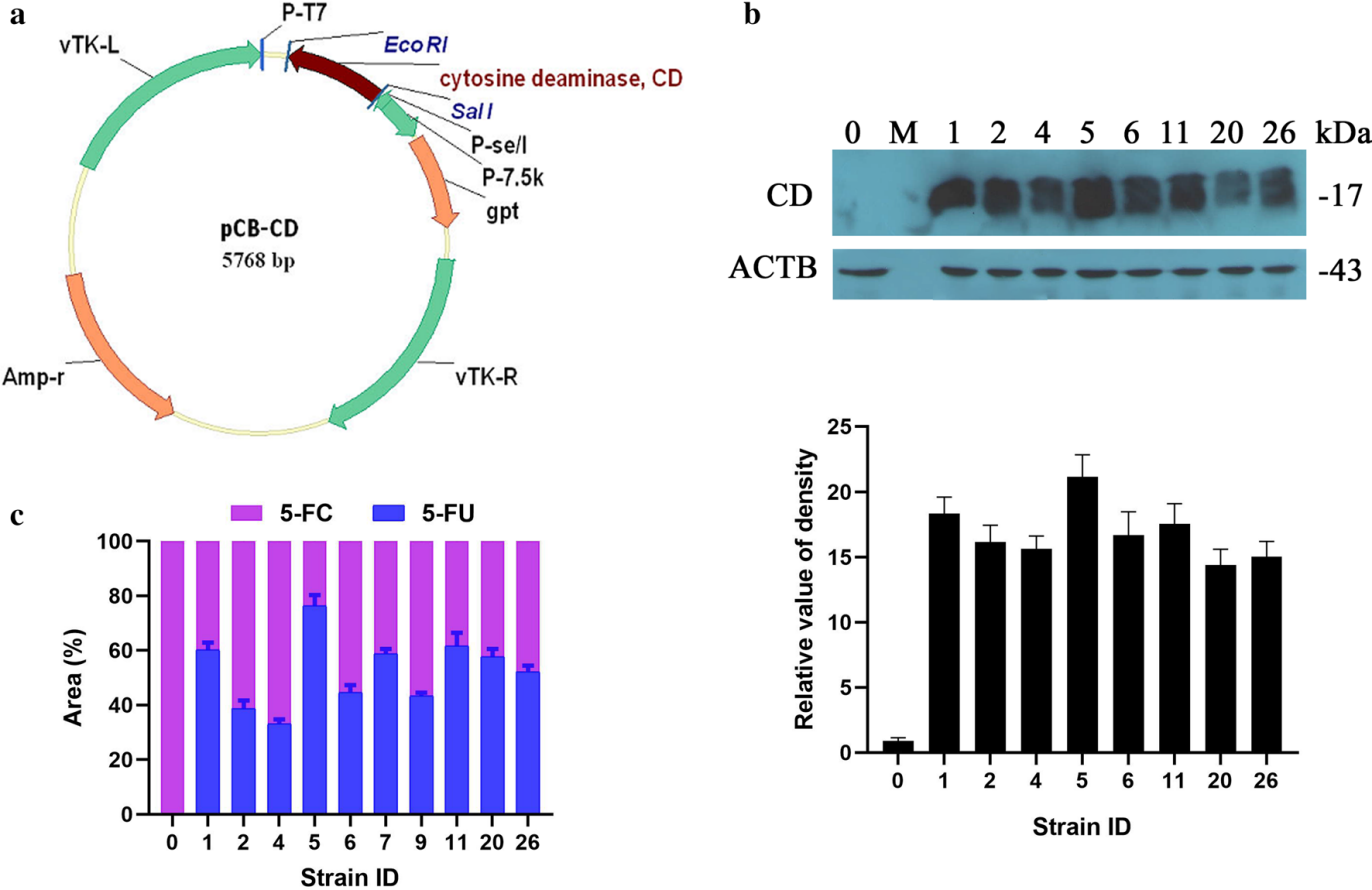
Characterization of vaccinia VG9-CD. **a** Schematic illustration of the shuttle plasmid pCB-CD. **b** Western blot proved the expression of CD in vaccinia VG9-CD recombinants. 0 represents vaccinia VG9-TK^−^ as a negative control. M represents protein Marker. Bottom graph showed relative CD levels that were normalized to ACTB. **c** CD activity determined by the conversion rate of 5-FC to 5-FU. Vaccinia lysates obtained from 10 vaccinia VG9-CD recombinants were added to 5-FC solution. The reaction run for 3 h at 37 °C, then the concentration of 5-FC and 5-FU were detected by the Waters Breeze HPLC unit. The conversion rate of 5-FC to 5-FU was calculated by comparing the proportion of peak area of 5-FU in the total peak areas of 5-FC and 5-FU

DNA sequence of yeast CD was synthesized according to GenBank accession No. NM_001184159. Three amino acid changes were introduced (A23L, I140L and V108I) to increase thermal stability of the yeast CD protein [30]. The vaccinia VG9 strain was obtained from the National Institutes for Food and Drug Control (NIFDC, Beijing 100050, China). The shuttle plasmid pCB used to generate vaccinia recombinants was provided by Professor Liu (Institute of Biochemistry and Cell Biology, Shanghai Institutes for Biological Sciences, The Graduate School, Chinese Academy of Sciences). pCB plasmid contains the flanking sequences of TK, which facilitated homologous recombination into the whole TK gene of vaccinia VG9. The synthetic yeast CD was placed under the control of the vaccinia synthetic early/late promoter P-se/l by cloning into the *Sal*I and *EcoR*I sites of pCB (Fig. 1a).

The recombinant pCB-CD plasmid was transfected into HEK-293 cells with Effectene^®^ Transfection Reagent (Qiagen, Germany), which had been infected with wild-type VG9 2 h before, then the CD gene with TK flanking sequences could replace the TK gene of the VG9 strain by homologous recombination. Recombinants were then selected in Vero cells in the presence of mycophenolic acid, xanthine, and hypoxanthine as xanthine-guanine phosphoribosyltransferase (XGPRT) selection. The vaccinia VG9-CD was plaqued for at least 6 rounds of selection to confirm that a pure recombinant stock was obtained. Screening recombinant was amplified in BSC-40 cells and purified over a sucrose gradient centrifugation. The titer of purified vaccinia VG9-CD was determined by plaque assay on Vero cells and expressed as plaque-forming units (PFU)/ml.

### Western blot for CD expression screening

10 strains of vaccinia VG9-CD purified by plaqued selection were propagated in BSC-40 cells. Cells and supernatants were harvested and lysed by 3 cycles of freezing and thawing. Vaccinia lysate were separated by 10% SDS-PAGE gel and transferred to a PVDF membrane (Life Technologies, NY, USA). The membrane was blocked with non-fat milk and incubated with anti-yeast CD polyclonal antibodies (2485–4906, Bio-rad, CA, USA) overnight at 4 °C. Finally, blots were developed with HRP-conjugated anti-goat IgG and detected by an ECL luminol reagent (Santa Cruz, CA, USA).

### HPLC analysis of CD activity

CD activity of vaccinia VG9-CD was measured by conversion of 5-FC to 5-FU [31, 32]. 1 ml CD enzyme reaction volumes contained 200 μl of vaccinia lysate and 800 μl of 1.25 mg/ml 5-FC in PBS. The reaction was typically allowed to run for 3 h at 37 °C, and 10 μl samples were removed for analysis by the Waters Breeze HPLC unit. 5-FC and 5-FU were detected using a solid-phase Inertsil ODS-3 column (5 μm, 4.6 × 250 mm, GL Sciences Inc., Japan) and a mobile phase that containing 95% 50 mmol/l ammonium phosphate with 0.01% tert-butylammonium perchlorate, pH 2.1 and 5% methanol. HPLC runs were at 1 ml/min mobile phase for 8 min. The Photodiode Detector Array (PDA) was set at 274 nm for 5-FC and 266 nm for 5-FU, respectively. The conversion rate of 5-FC to 5-FU was calculated by comparing the proportion of peak area of 5-FU in the total peak areas of 5-FC and 5-FU.

### In vitro cytotoxicity assay

The colon adenocarcinoma cell lines HCT 116, CT26, MC38 and other cancer cell lines were seeded into 96-well plates at 1 or 2 × 10^4^ cells/well. After overnight growth, cells were incubated in fresh growth medium with or without 0.1 mg/ml 5-FC and infected with VG9-CD at different multiplicity of infections (MOIs) (0, 0.01, 0.1, 1, 2 and 10). 48 h after infection, 10 μl of 3-(4,5-dimethylthiazol-2-yl)-2,5-diphenyltetrazolium bromide (MTT) solution was added to each well, and the cells were incubated at 37 °C for 4 h. The supernatant was removed by vacuum and replaced by 150 μl dimethylsulfoxide. Complete solubilization of formazan crystals was achieved by 10 min of shaking at room temperature. Then, the plates were read on a SpectraMax M5 Multi-Mode Microplate Reader (Molecular Devices, CA, USA) at 490 nm. The relative viability (% of control) was calculated as follows: (absorbance of experimental samples–background absorbance)/(absorbance of untreated controls–background absorbance) × 100%.

### Animal experiments

To establish carcinoma tumor models, the immunocompromised nude mice and immunocompetent C57BL/6 and BALB/c mice were subcutaneously implanted with 2 × 10^6^ HCT116, MC38 or CT26 cells on left oxter. Mice were 4–6 weeks old for all studies. The numbers of male and female mice in each experimental group were matched. After 4–10 days of tumor growth, the tumors reached the size of 3–5 mm in diameter. Then, 1 × 10^7^ PFU of purified VG9, VG9-TK^−^ or VG9-CD, which suspended in 100 μl of PBS, were intratumorally injected into mice. For co-administration, fluorocytosine tablets were dissolved in PBS, centrifuged for supernatant containing 5-FC, and intratumorally injected or intragastric administered into tumor bearing mice at the dose of 500 mg/kg/day. 5-FC treatment is daily performed and lasted 4 weeks. Tumor volume (mm^3^) was calculated by the formula (L × H × W)/2, where L is the length, W is the width, and H is the height of the tumor in millimeters. Tumor growth was monitored weekly. Mice were euthanized 60 days after virus injection and the survival curves were plotted.

### Pharmacokinetic analysis

Tumors on C57BL/6 mice were isolated on day 1, 3, 5, 7, 14, and 21 for pharmacokinetic analysis of 5-FC and 5-FU 2 h after the last dose of 5-FC. Tumors were homogenized in 1 ml PBS using an IKA T10 basic ULTRA-TURRAX. The homogenates were lysed by 3 cycles of freeze–thaw process, and centrifuged for 5 min at 3000*g* at 4 °C. Then, quantitative determination of 5-FC and 5-FU was conducted by HPLC analysis as previously described. Meanwhile, viral titers of the remaining supernatants were determined by plaque assays on BSC-40 cells.

### Tumor rechallenge

Tumor rechallenge was performed on 5 cured BALB/c mice from subcutaneous CT26 tumor long-term survival studies. The cured mice were subcutaneously reimplanted on the opposite flank with CT26 cells at the dose of 2 × 10^6^ and monitored for tumor growth twice a week. Age-matched control mice were also challenged.

### Statistical analysis

Survival data was plotted by the Kaplan–Meier method, and differences between curves were compared by the log-rank test. GraphPad Prism software was used for all statistical analysis.

## Results

### Characterization of vaccinia VG9-CD

The recombinant vaccinia VG9-CD was generated from attenuated Tian Tan vaccinia strain Guang9 and replaced TK gene of the VG9 by yeast CD gene with the shuttle plasmid pCB-CD. The plasmid profile of pCB-CD was shown in Fig. 1a. 10 strains of purified recombinants were obtained by 6 rounds of plaqued selection. To confirm CD protein was correctly expressed, western bolt assay was applied and the level of CD expression in vaccinia VG9-CD recombinants was shown in Fig. 1b. The results suggested that all recombinants expressed yeast CD as expected, while No. 1 and No. 5 had the highest expression level. Furthermore, the activity of CD, that is, the ability of 5-FC to convert to 5-FU, was determined by HPLC. Figure 1c showed the conversion rate of 5-FC to 5-FU between 10 vaccinia recombinants and results revealed that No. 1 and No. 5 had the highest conversion ability. Taken together, we chose No. 5 as the origin strain and amplified for further study.

### Oncolytic potency of vaccinia VG9-CD in vitro

**Fig. 2:**
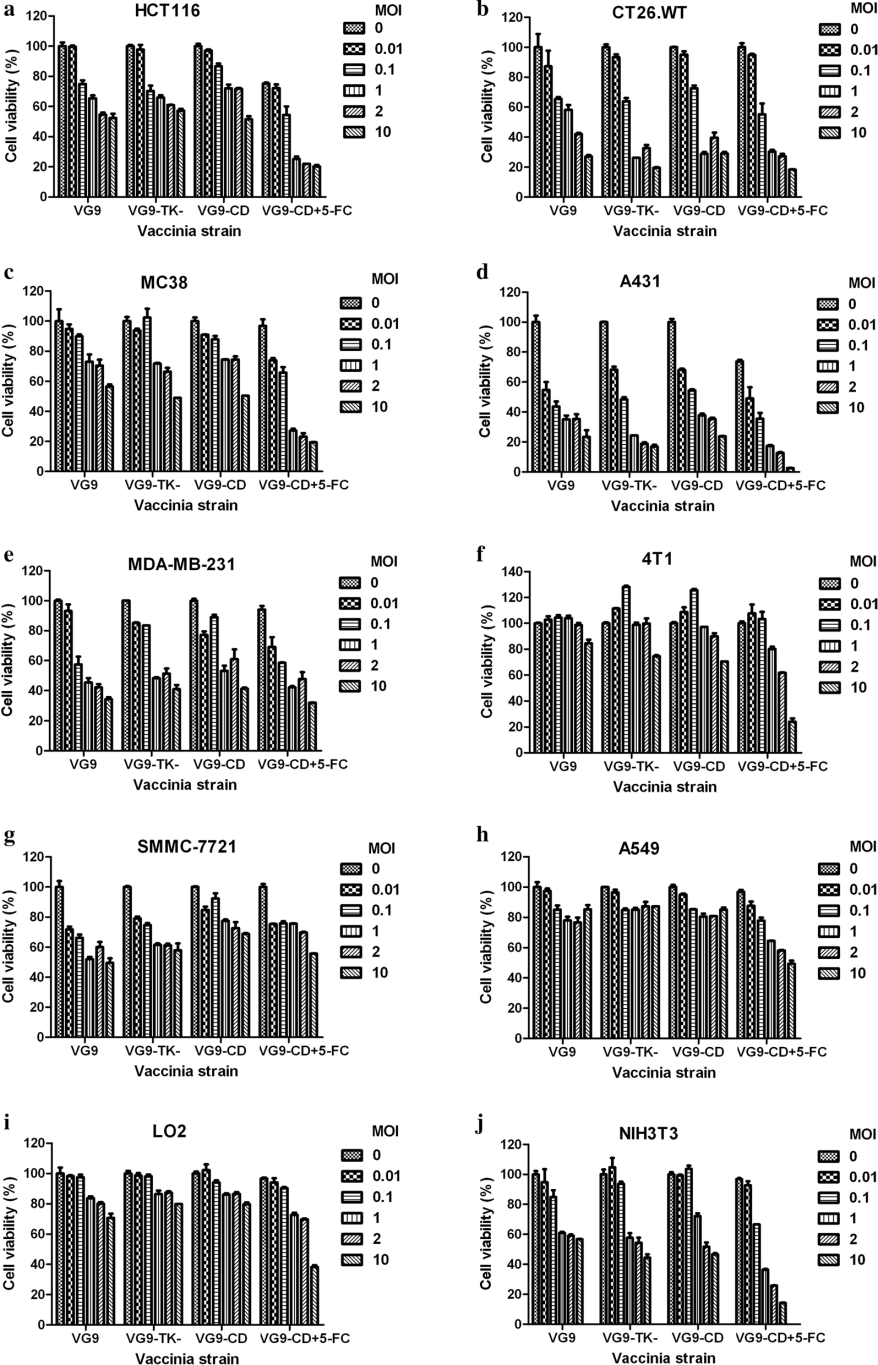
In vitro cytotoxicity effect of vaccinia VG9-CD. Cancer cell lines of HCT 116 (**a**), CT26.WT (**b**), MC38 (**c**), A431 (**d**), MDA-MB-231 (**e**), 4T1 (**f**), SMMC-7721 (**g**), A549 (**h**) and normal cells of LO2 (**i**), NIH3T3 (**j**) were infected with increasing titers of VG9, VG9-TK- and VG9-CD (MOI = 0, 0.01, 0.1, 1, 2 and 10) for 48 h. 0.1 mg/ml 5-FC was added to cells infected with vaccinia VG9-CD. Cell viability was measured by MTT assay. Each bar represents the mean ± SD (n = 3)

Various cancer cell lines were infected with increasing titers of VG9, VG9-TK^−^ and VG9-CD in order to determine the oncolytic potency of vaccinia in vitro. It is illustrated that VG9 was the wild-type of vaccinia, and VG9-TK^−^ was TK deleted vaccinia without any gene expressed. As shown in Fig. 2, all viruses, including VG9, VG9-TK^−^ and VG9-CD, exhibited high oncolytic activity on colon adenocarcinoma cell lines of HCT 116, CT26 and MC38, and there was no significant difference among the three vaccinia viruses. For other cancer cells, viruses showed different oncolytic potency. Results revealed that vaccinia viruses displayed good oncolytic ability on A431 and MDA-MB-231 cells, but showed poor oncolytic activity on 4T1, SMMC-7721 and A549 cell lines. In addition, viruses exhibited different oncolytic ability on normal cells, good on NIH3T3 and poor on LO2. In case of 5-FC addition with VG9-CD virus, the oncolytic effect was significantly increased compared to VG9-CD alone, indicating the synergetic effect of 5-FU and vaccinia virus. Moreover, we observed that the synergetic effect was more effective when the titers of VG9-CD reached a certain level, 1 MOI at the least and 10 MOI most effective. It is suggested that only when a certain concentration of CD protein is reached, can the conversion efficiency of CD be brought into play.

### Antitumor efficacy of vaccinia VG9-CD in vivo

**Fig. 3:**
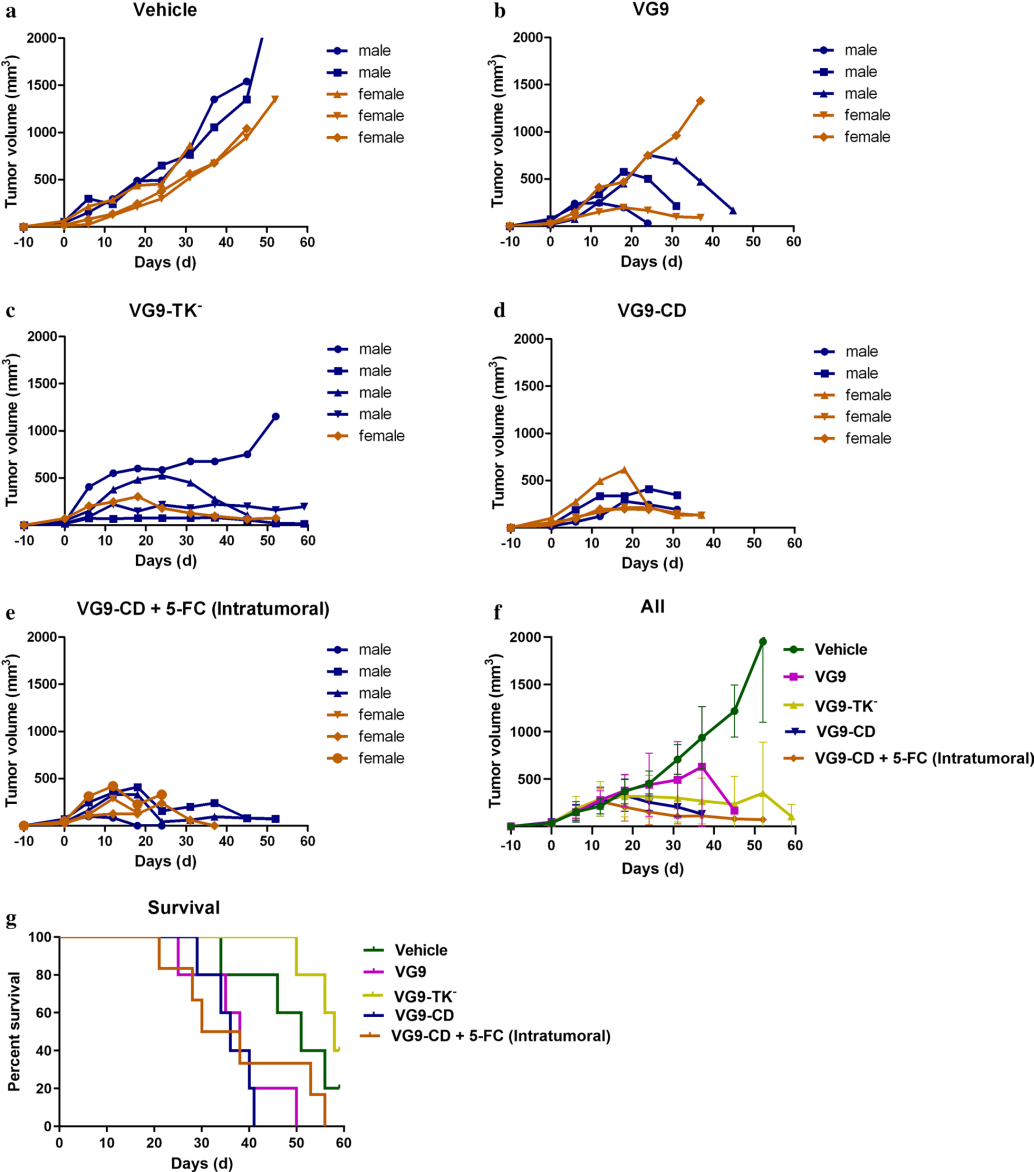
Antitumor efficacy of vaccinia VG9-CD in nude mice bearing HCT116 colon cancer cells. **a**–**e** Tumor volumes in nude mice treated with PBS (**a**), VG9 (**b**), VG9-TK^−^ (**c**), VG9-CD (**d**) and VG9-CD in combination with intratumoral administration of 5-FC (**e**). **f** Average tumor volumes of all the treatment. **g** Kaplan–Meier survival curves for tumor bearing nude mice treated with vaccinia viruses

To investigate the antitumor efficacy of VG9-CD in colorectal cancer, the human colon carcinoma cell line of HCT116 was established in nude mice model. Mice were intratumorally injected with PBS, VG9, VG9-TK^−^ and VG9-CD after 10-day of tumor growth, then tumor volume and survival were followed up for 59 days. The prodrug 5-FC was intratumorally administered at the dose of 500 mg/kg/day in mice receiving VG9-CD treatment and lasted 4 weeks. As shown in Fig. 3, all the viruses, including VG9, VG9-TK^−^ and VG9-CD, significantly suppressed tumor progression as expected. However, the survival rate of mice subjected viral infection was quite low, except VG9-TK^−^. Mice treated with wild-type VG9 died earlier than those treated with PBS, though the tumor growth was partially suppressed. The same situation was occurred in VG9-CD treated mice, but with better tumor remission. Moreover, if vaccinia VG9-CD is combined with 5-FC prodrug, tumor progression was more effectively regressed. Of all 6 treated mice, complete clearance of the tumor was confirmed in 2 mice, but both of them were dead finally. To our surprise, VG9-TK^−^ treated mice showed the best tumor remission ability, 2 mice finally survived and showed tumor stabilized or regressed.

**Fig. 4:**
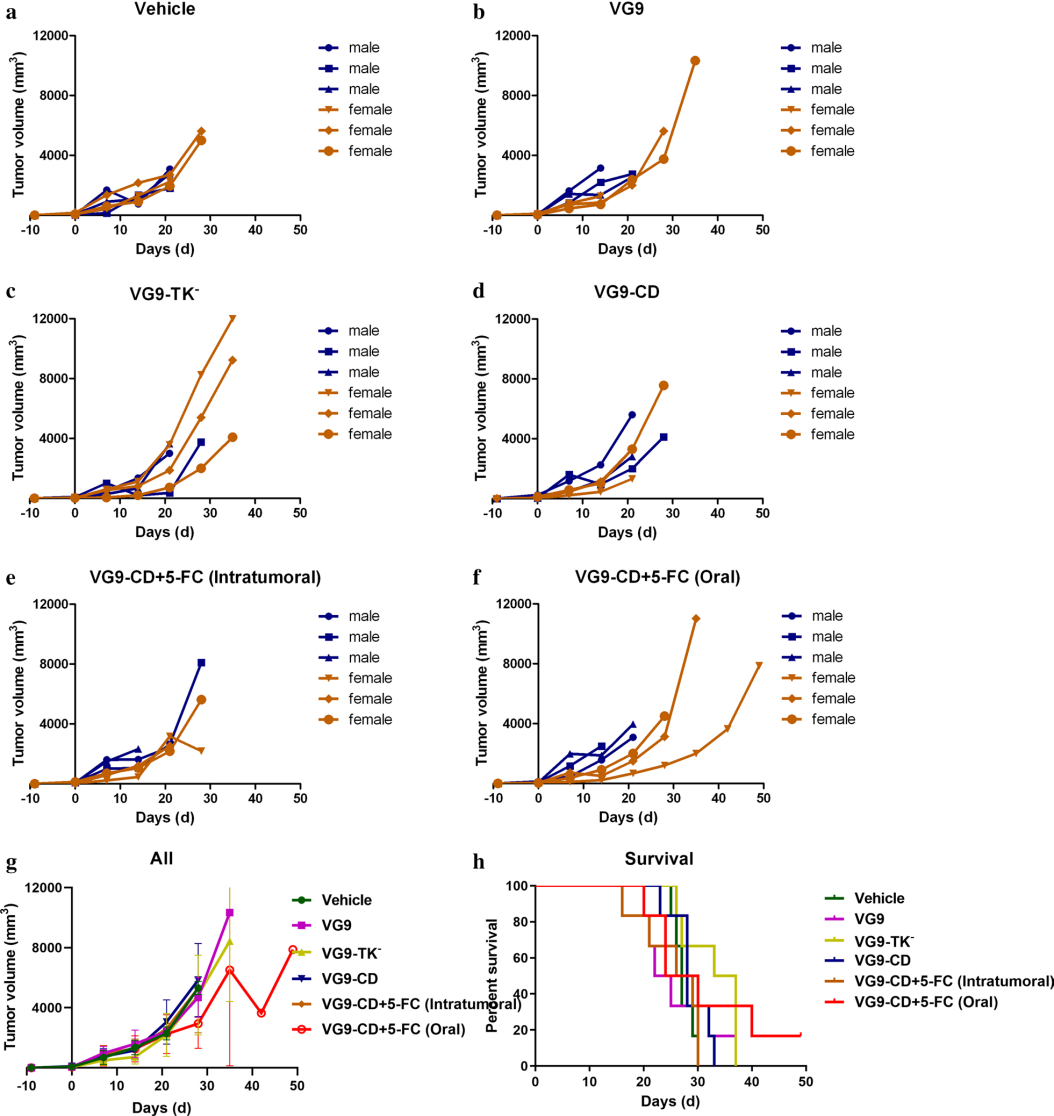
Antitumor efficacy of vaccinia VG9-CD in C57BL/6 mice bearing MC38 colon cancer cells. **a**–**f** Tumor volumes in C57BL/6 mice treated with PBS (**a**), VG9 (**b**), VG9-TK^−^ (**c**), VG9-CD (**d**), VG9-CD in combination with intratumoral administration of 5-FC (**e**) and VG9-CD in combination with gavage administration of 5-FC (**f**). **g** Average tumor volumes of all the treatment. **h** Kaplan–Meier survival curves for tumor bearing C57BL/6 mice treated with vaccinia viruses

The immunotherapy effect of vaccinia virus could not be exhibited in immune-deficient nude mice, so colorectal cancer models in immunocompetent C57BL/6 and BALB/c mice were established to investigate the antitumor effect of vaccinia VG9-CD. Daily injection of prodrug 5-FC destroyed the integrity of tumors, which enhanced the risk of external infection in mice. As a result, oral gavage administration of 5-FC was tried in the next experiments. C57BL/6 mice were given subcutaneous implantation of MC38 cells followed by an intratumoral injection of vaccinia viruses. Combination administration of 5-FC lasted for 4 weeks and the mice were observed for 3 additional weeks without further treatment. As shown in Fig. 4, vaccinia viruses were failure to suppress tumor progression, however, they prolonged survival of tumor bearing mice compared to PBS treated mice. No tumor remission occurred in VG9 and VG9-CD group, while the survival rates were only slightly improved. In combination treatment of VG9-CD and 5-FC, the results depend on the way of 5-FC is administered. Vaccinia VG9-CD combined with daily gavage administration of 5-FC significantly prolonged the survival of tumor bearing mice, though the tumors still kept growing. However, VG9-CD combined with daily intratumoral injection of 5-FC failed to improve the survival status of C57BL/6 mice, worsened on the contrary. Consistent with the results of nude mice, the therapeutic effect of VG9-TK^−^ was better than that of the other two viruses.

**Fig. 5:**
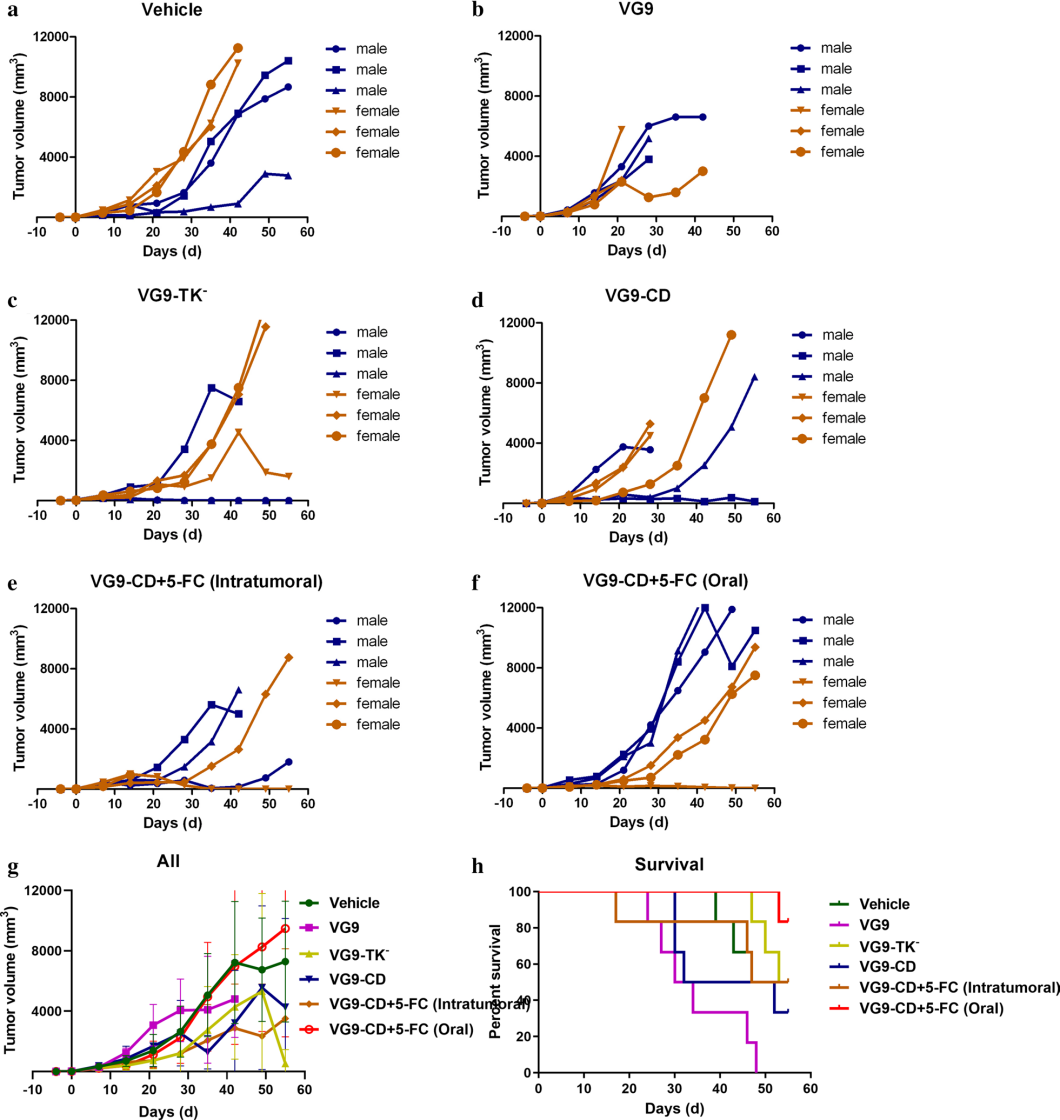
Antitumor efficacy of vaccinia VG9-CD in BALB/c mice bearing CT26.WT colon cancer cells. **a**–**f** Tumor volumes in BALB/c mice treated with PBS (**a**), VG9 (**b**), VG9-TK^−^ (**c**), VG9-CD (**d**), VG9-CD in combination with intratumoral administration of 5-FC (**e**) and VG9-CD in combination with gavage administration of 5-FC (**f**). **g** Average tumor volumes of all the treatment. **h** Kaplan–Meier survival curves for tumor bearing BALB/c mice treated with vaccinia viruses

Another genus of immunocompetent BALB/c mice was subcutaneously inoculated with CT26 colon cancer cells. Tumors were allowed to grow for 4 days until the average tumor size reached 3–5 mm in diameter. Then, vaccinia viruses were intratumorally injected and followed up for 55 days. Results showed the wild-type VG9 was most cytotoxic, as it induced the death of mice (Fig. 5). Meanwhile, TK deletion minimized the cytotoxicity of VG9, as VG9-TK^−^ showed better survival status and partial tumor regression compared to PBS treated mice. Expression of exogenous protein increased the cytotoxicity of VG9-TK^−^, for the survival rate of VG9-CD was poor than VG9-TK^−^ and PBS groups but better than VG9. Moreover, in combination with gavage administration of 5-FC, VG9-CD showed the best survival status though the tumor regression was not significant. Better survival and tumor growth remission were observed in VG9-CD combined with intratumoral injection of 5-FC than VG9-CD alone, but it was poor than oral gavage group. Except for VG9 group, all viral treatment groups have one mouse cured at least, of which 2 in VG9-TK^−^ group.

### Pharmacokinetic of vaccinia VG9-CD in tumor tissues

**Fig. 6:**
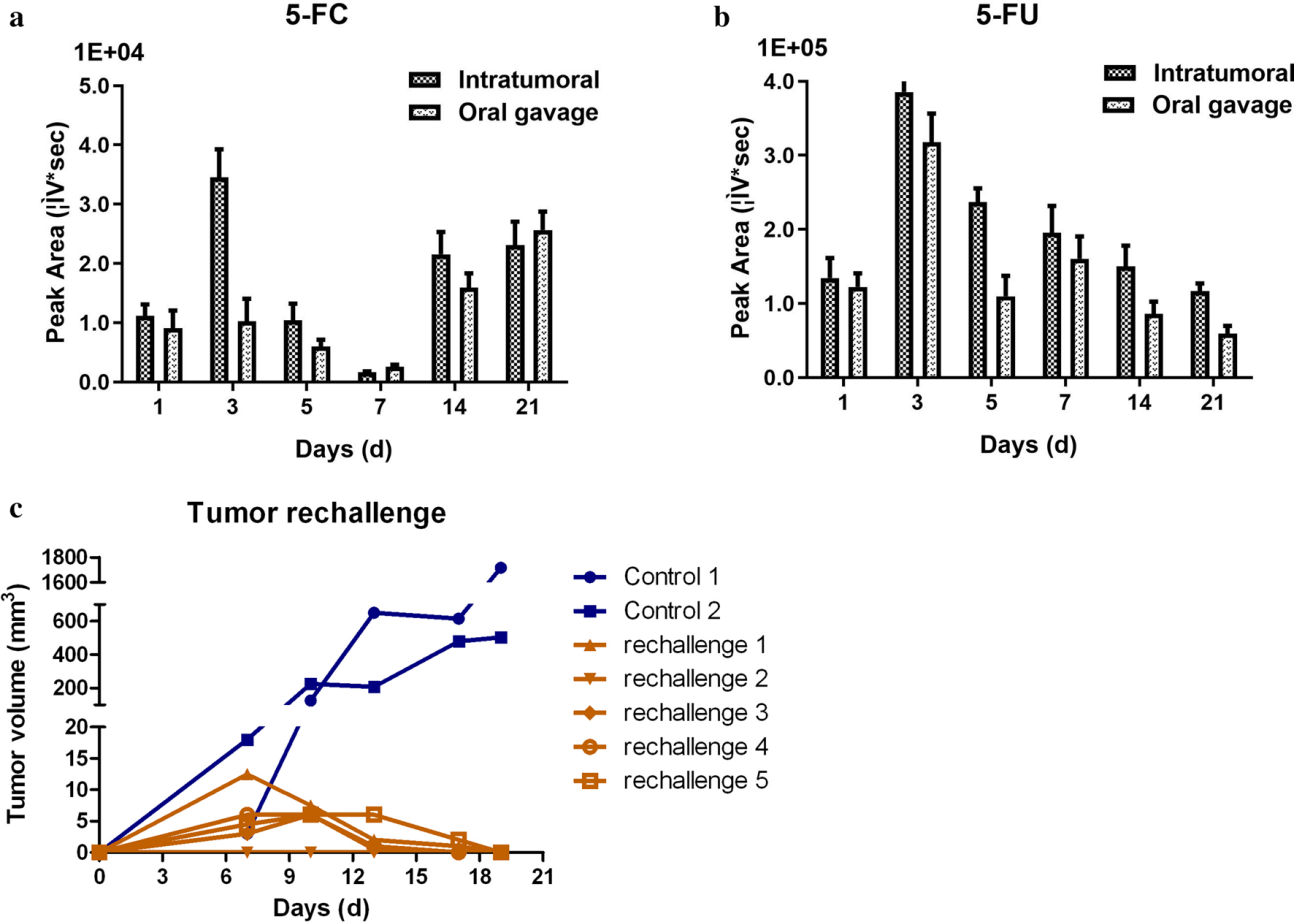
**a**, **b** Quantitative determination of 5-FC (**a**) and 5-FU (**b**) by HPLC analysis. **c** Tumor volumes in previously cured BALB/c mice subcutaneously rechallenged with CT26.WT cells

Efficient intratumoral conversion of 5-FC prodrug into the anticancer drug 5-FU was detected after VG9-CD infection combined with intratumoral or oral administration of 5-FC. As shown in Fig. 6a, b, mice given VG9-CD and 5-FC treatment had comparable levels of 5-FU and low levels of 5-FC, among which the concentration of 5-FC is one order of magnitude less than that of 5-FU. Results showed that the levels of 5-FU were relatively high in the first week and decreased in the following 2 weeks. Meanwhile, the levels of 5-FC were on the contrast. In addition, the levels of 5-FU were highest on day 3 after virus infection. These results suggested that the optimized CD gene efficiently converted most of the available 5-FC to 5-FU in the tumor and the process lasted for a long time. Furthermore, the remaining supernatants were used to determine viral titers by plaque assays. Table 1 revealed that viral titers of isolated tumors were very high after intratumoral injection of VG9-CD, regardless the administration mode of 5-FC. The highest viral titers appeared on day 5. Even 3 weeks after virus injection, the viral titers of VG9-CD were still high.

**Table 1:** pharmacokinetic of viral titers by vaccinia VG9-CD in tumor tissues of C57BL/6 mice

Days (d)	Plaque assay (PFU/ml)	
	Intratumoral	Oral gavage
1	8.7 × 10^3^	5.6 × 10^3^
3	1.4 × 10^4^	9.6 × 10^3^
5	2.9 × 10^5^	1.3 × 10^5^
7	7.6 × 10^3^	5.4 × 10^3^
14	3.4 × 10^3^	2.8 × 10^3^
21	1.2 × 10^4^	5.8 × 10^4^

### Tumor rechallenge

As mentioned earlier, 5 BALB/c mice bearing CT26 tumor cells were cured by vaccinia therapy. To evaluate whether antitumor immunity had developed in immunocompetent BALB/c mice, 5 cured mice were rechallenged on the opposite flank with CT26 cells the same dose as the first inoculation. As shown in Fig. 6c, tumors successfully engrafted in 2 age-matched control mice, which had not previously been exposed to CT26 tumors. However, the previously cured mice were in contrast. Although subcutaneous tumors were observed initially on day 7 after rechallenge, these tumors all disappeared spontaneously by day 19 without recurrence.

## Discussion

Gene-directed enzyme/prodrug therapy (GDEPT), which converts a systemically administered non-toxic prodrug into high local concentration of an active anticancer drug in tumors, is an alternative therapeutic method to conventional chemotherapy [21, 33]. The system involves the activation of a prodrug by an enzyme, and requires a vehicle for the enzyme gene delivery [34]. In particular, CD derived from bacteria or yeast combined with 5-FC has demonstrated a sufficient antitumor effect for several types of cancers [33, 35, 36]. Additionally, vaccinia Tian Tan strain is an effective viral vector for delivery of a prodrug-activating enzyme to specific tumor regions due to its improved ability of tumor tropism [17, 18].

In this study, a TK deleted VG9 strain with active yeast CD expression was constructed, and the characterization of CD including CD antigenicity and high efficacy of conversion 5-FC to 5-FU was verified by western blot and HPLC analysis. Then, the in vitro oncolytic potency of vaccinia VG9-CD was evaluated in various cancer cells and normal cells. Results showed that vaccinia viruses displayed different oncolytic potency in vitro cells, no relationship with whether they were cancer cells or normal cells. Theoretically, vaccinia viruses preferentially infect and lyse proliferating cells, such as cancer cells, and not cause excessive damage to resting cells, such as normal tissues. However, the situation of cells in vitro was very different from the cells grown in vivo environment. In vitro cells were detached from the overall domination and regulation of the body, and their growth depends on the nutrients and cell density of the environment. Thus, the division rate of cancer or normal cells in vitro determines the oncolytic efficacy of vaccinia virus. Besides, the morphology and other cellular characteristics of in vitro cells were also related to the oncolytic ability of vaccinia viruses.

The combination of VG9-CD and 5-FC plays combined effect of vaccinia virus and chemotherapy, which results in augmented antitumor effects. Vaccinia viruses mediate tumor regression through their potent intrinsic oncolytic effect and activation of antitumor immune responses, while 5-FU converted by VG9-expressed CD directly kills tumor cells. In all three colorectal tumor models, vaccinia viruses VG9 and VG9-CD showed great cytotoxicity and shortened the survival of mice. However, VG9-TK^−^ showed excellent survival and tumor regression, suggested that TK deletion minimized the cytotoxicity of VG9 while exogenous expression elevated the toxicity of virus again. The addition of 5-FC improved the therapeutic effect of VG9-CD, but the improvement of effect is related to the administration of 5-FC. Daily intratumoral injection of 5-FC leads to tumor destruction, making the mice susceptible to external infections and shortening the survival. By contrast, oral gavage administration avoids the problem and showed the best therapeutic effect, even better than VG9-TK^−^ group in both immunocompetent mice models.

Besides direct killing of tumor cells, stimulation of host immune responses by oncolytic virus infection provides long lasting antitumor activity and results in clearance of the tumor [37]. We performed tumor rechallenge in 5 cured BALB/c mice and found regenerative tumors recovered spontaneously. It is indicated that antitumor immune response was activated in virotherapy and enhanced the eradication of tumors. In addition, we found the survival of tumor bearing C57BL/6 mice was far shorter than that of BALB/c mice. MC38 tumors grew fast in C57BL/6 mice, and the mice died naturally within 30 days. Whereas, the rapid growth of CT26 tumors in BALB/c mice occurred 30 days after implantation and lasted for the whole survival period. It is supposed the immunity of the two mouse models was different, and BALB/c mice had a stronger and active immune system.

## Conclusions

A TK deleted VG9 strain with active yeast CD expression was constructed and the antitumor efficacy of VG9-CD in combination with 5-FC prodrug in colorectal cancer models was evaluated. To our surprise, VG9-TK^−^ showed the best effect in vaccinia only therapy, represented by partial tumor remission and prolonged survival. However, with the synergistic effect of 5-FC, especially by gavage administration, vaccinia VG9-CD exhibited excellent antitumor efficacy even better than VG9-TK^−^, prolonged survival in particular. Therefore, we conclude that vaccinia VG9-CD in combination with 5-FC plays combined effect of vaccinia virus and chemotherapy, and becomes a promising therapeutic method for cancer treatment.

## Data Availability

The data that support the findings of this study are available from the corresponding author upon reasonable request.

## Abbreviations

TK: Thymidine kinase
5-FU: 5-fluorouracil
5-FC: 5-fluorocytosine
CD: Cytosine deaminase
VG9: Vaccinia virus Guang 9
PFU: Plaque-forming units
HPLC: High-performance liquid chromatography
MOI: Multiplicity of infection
